# Associated data on the physicochemical properties of pedosediments, climatic and dendrochronological indicators for palaeogeographic reconstructions

**DOI:** 10.1016/j.dib.2019.104829

**Published:** 2019-11-16

**Authors:** Fedor Lisetskii, Arseniy Poletaev, Evgenia Zelenskaya, Vitaliy Pichura

**Affiliations:** a Federal and Regional Centre for Aerospace and Surface Monitoring of the Objects and Natural Resources, Belgorod National Research University, Belgorod, 308015, Russian Federation; b Institute of Earth Sciences, Belgorod National Research University, Belgorod, 308015, Russian Federation; c Kherson State Agrarian University, Kherson, 73006, Ukraine

**Keywords:** Pedosediments, Macroelements, Trace elements, Precipitation, Tree ring chronologies

## Abstract

Palaeogeographic markers can be justified among a large number of geochemical indicators in separate layers of pedosediments. This determines the need to develop a system of most information-rich pedogenetic indicators for reconstruction of the dynamics of erosion-accumulative processes based on dated earthen defensive constructions of the historical period. We demonstrated this solution at the example of a frontier rampart with a ditch of the mid-17th century (“Catena linking of landscape-geochemical processes and reconstruction of pedosedimentogenesis: a case study of defensive constructions of the mid-17th century, South Russia” [1]). Using individual of macroelements and trace elements as part of complex geochemical relationships and indicators allows us to determine the geochemical associations of elements that diagnose migration of the sediments at the *trans*-eluvial catenas. It is shown that in the forest-steppe conditions the determined system of pedogenic indicators, such as content of particles (%) with the size >0.01 and < 0.005 mm, pH_H2O_, CO_2,_ (CaО+MgO):Al_2_O_3,_ Si:Al, CaO:TiO_2,_ the eluviation coefficient, the association of mobile (Ca, Na, Mg, Sr) and weakly mobile (K, Ba, Rb) elements, the sum of the elements accumulated in the soil (P, Ca, K, Mg, Mn, Cu). They can be the basis of this classification and chronostratigraphy of pedosediments. The data obtained in these investigations are aimed at the establishment of soil-geomorphologic interrelations and calibration of mathematical models of natural processes.

**Table:** Specifications Table

Subject area	Soil Science
More specific subject area	Geochemistry of the landscape, Palaeogeography
Type of data	Table Image Figure
How data were acquired	Laser analyser of the size of particles Analysette 22 MicroTec (Fritsch GmbH). X-ray fluorescence spectrometer (Spectroscan Max-GV, ‘SPECTRON’, Ltd). pH values (H_2_O) were determined by a potentiometric method (pH meter Sartorius Basic Meter PB–11). LINTAB™ 6 and TSAP-Win (Rinntech, Heidelberg, Germany). STATISTICA Advanced + QC for Windows v.10 Ru and STATISTICA Automated Neural Networks for Windows v.10 Ru.
Data format	Raw
Parameters for data collection	Data was collected on the rampart slope (points 1–3) and along the thalweg of the defensive ditch at 270 m (point 4) and farther at 160.7 m (point 5) and the vertical profile of the pedosediments in the closing alignment of the ditch. A study in 2013 made it possible to isolate 8 layers in the profile of the pedosediments, and after verification, using the results of palaeogeographic reconstruction, 10 layers with more accurate boundaries (chronozones) were identified in 2019.
Description of data collection	Determination of the gross composition of soils in general and particles <1 mm, the values of geochemical ratios and coefficients, as well as their distribution along the profile of pedosediments, a system of most information-rich pedogenetic indicators for palaeogeographic reconstruction. Data on the annual amount of precipitation and tree-ring growth of the oak-tree that were used for climate reconstruction are presented.
Data source location	Belgorod Oblast, Russia. ‘Karpov section of the Belgorod Line’ (Catena on the slope of the rampart: 50° 39′ 51.9″ N; 36° 23′ 06″ E; Mouth of the ditch: 50° 39′ 43.3″ N; 36° 23′ 23.5″ E). Meteorological station ‘Bogoroditskoye-Fenino’ (51° 9′ 40″N 37° 21′ 11″E). Plot ‘Forest on the Vorskla’ (State Reserve ‘Belogorye’) (50° 36′ 58″N 35° 58′ 1″E).
Data accessibility	Raw data was deposited at repository Mendeley Data under Identification number: w9jtszpmst.1; Aug 16, 2019. Direct URL to data: https://doi.org/10.17632/w9jtszpmst.1
Related research article	F.N. Lisetskii, V.I. Pichura, Catena linking of landscape-geochemical processes and reconstruction of pedosedimentogenesis: a case study of defensive constructions of the mid-17th century, South Russia, Catena. (2020). https://doi.org/10.1016/j.catena.2019.104300 [1]

**Table:** 

**Value of the Data** • The data obtained here will contribute to our understanding of how climatic periods (beyond instrumental observations) changed the rate of accumulation of pedosediments. • A set of geochemical relationships and coefficients that were not sensory enough to reflect the intra-century rhythm of sedimentogenesis are presented in order to increase the effectiveness of such studies in the future. • The data in the form of a system of most information-rich pedogenetic indicators could be used for reconstruction of the dynamics of erosion-accumulative processes and calibration of mathematical models of these processes. There is a great information potential in the application of interdisciplinary methods in sedimentation studies, particularly those based on the data of field explorations (stratigraphic, sedimentological and micromorphological studies), geochemical features and dendrochronology [[2], [3], [4], [5], [6]].

## 1 Data

**Fig. 1:**
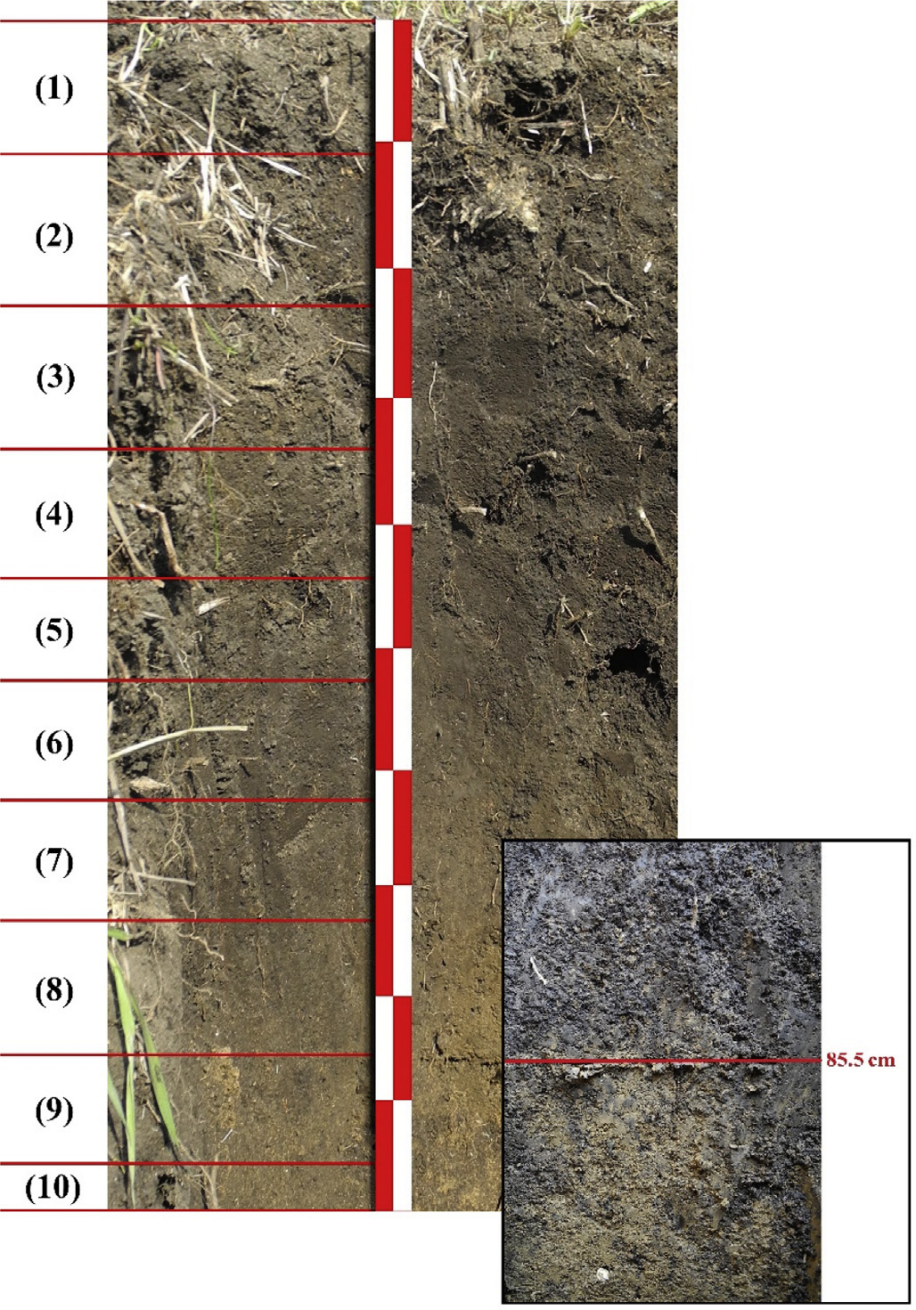
New (2019) vertical profile of pedosediments and their lower border at a depth of 85.5 cm. Layer numbers correspond to the depths: 0–11, 11–23, 23–34, 34–44, 44–52.5, 52.5–62.5, 62.5–73, 73–85.5, 85.5–96, 96–100 cm.

The method of obtaining data of the rate of soil erosion and accumulation uses the information capabilities of the earthen fortifications (catena on a frontier rampart with a ditch) as soil-formation models [7]. In order to identify the physicochemical and geochemical features we have collected data on the granulometric composition, concentration of macroelements and trace elements (25 metals and oxides) in soils and in particles with the size <1 mm, fractional composition of the humus were analysed in soil samples taken from different points in the catena and along the vertical profile of the pedosediments for the original (2013) [1] and verified (2019) options (Fig. 1). The vertical position of the layers was different: 0–10, 10–20, 20–30, 30–50, 50–70, 70–90, 90–95, 95–100 cm (2013); 0–11, 11–23, 23–34, 34–44, 44–52.5, 52.5–62.5, 62.5–73, 73–85.5., 85.5–96, 96–100 cm (2019). Climate data were obtained from the archive of the Bogoroditskoye-Fenino meteorological station named after I.A. Pulman, which is a reference for the Hydrometeorological Centre of Russia, since it is one of the oldest (founded in 1881) and its location has not changed. For reconstruction of the conditions of moistening was used dendrochronological data. This is a saw cut of oak (*Quercus robur*) aged 224 years, which was obtained in 1968 within the frame of the International Biological Program, IBP (the sample is kept in the museum of the environment of the preserve ‘Belogorye’). The tree grew in an oak-grove 250–300 years old in the southeast of the plot ‘Forest on the Vorskla’ (29 km from the Karpov rampart).

**Fig. 2:**
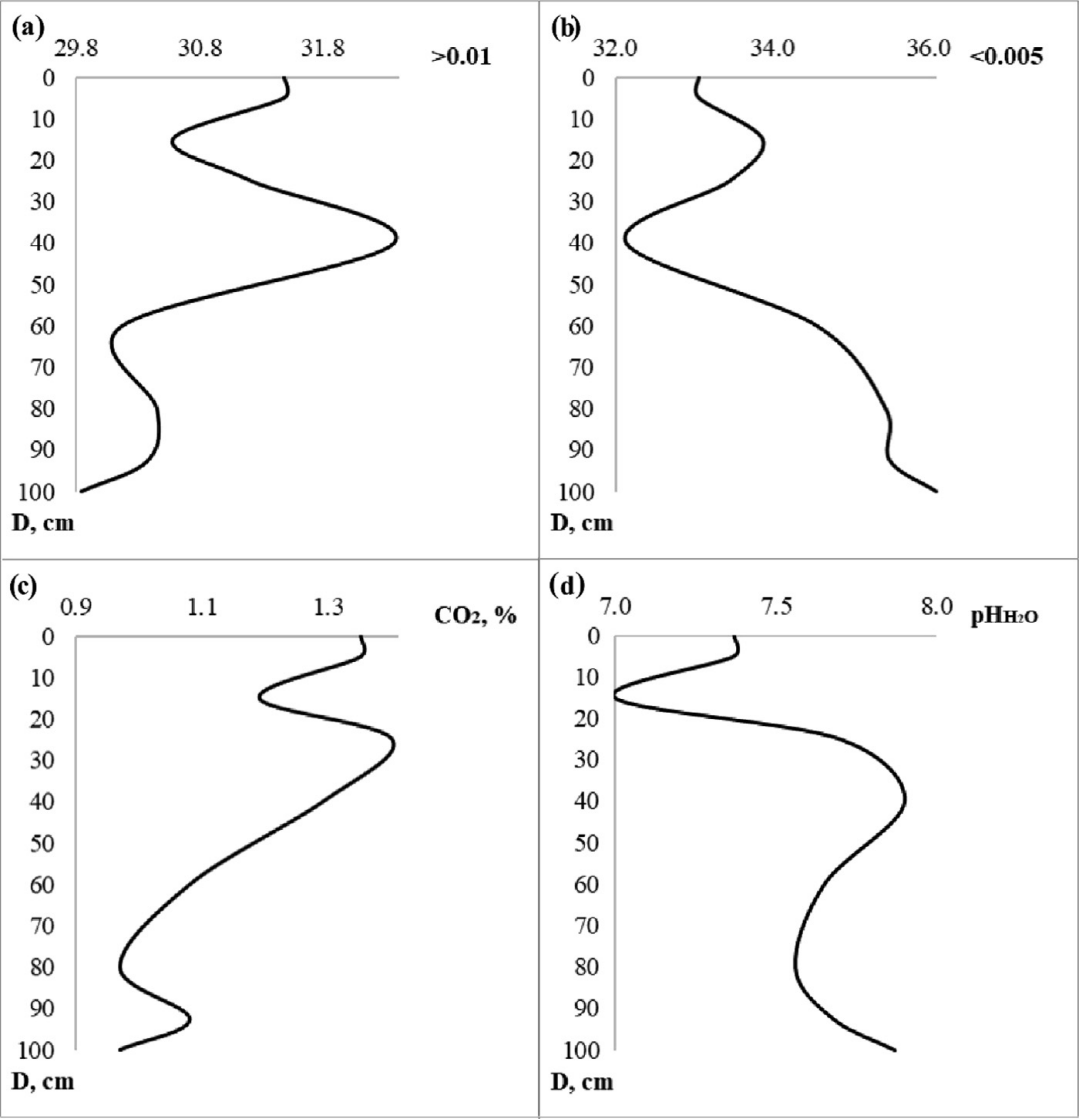
Distribution by profile of pedosediments of the main physico-chemical parameters (2013).

**Fig. 3:**
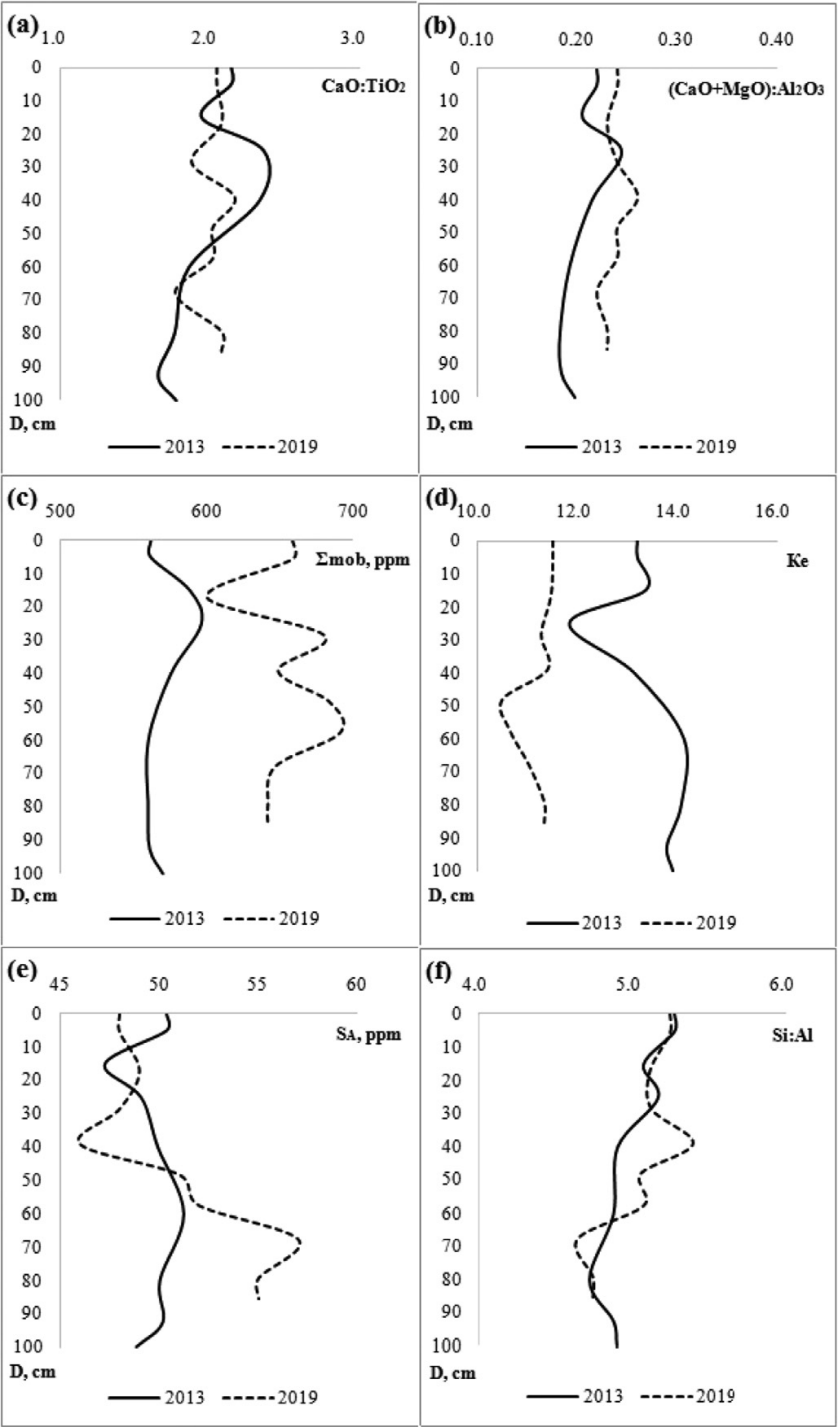
Distribution by profile of pedosediments of the geochemical relationships and coefficients (2013 and 2019).

**Fig. 4:**
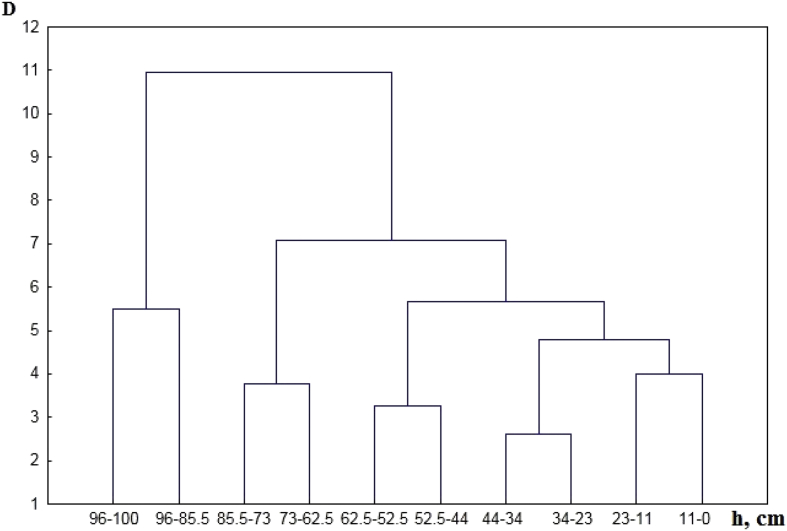
The results of a cluster analysis based on the concentration of chemical elements and oxides that were used to calculate geochemical coefficients (SiO_2_, Al_2_O_3_, CaO, MgO, P_2_O_5_, K_2_O, MnO, Cu, Na, TiO2, Sr, Ba, Rb) in the layers of pedosediments (2019).

**Fig. 5:**
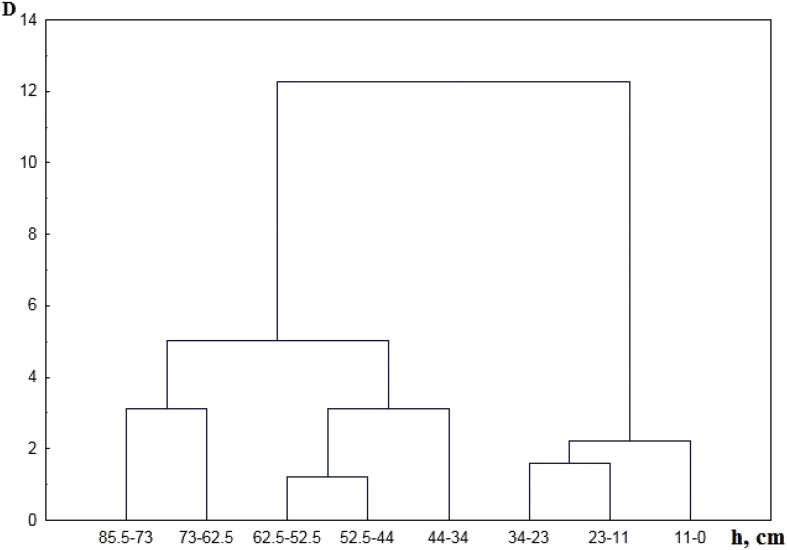
The results of cluster analysis by the values of 10 geochemical coefficients in the layers of pedosediments (2019).

**Fig. 6:**
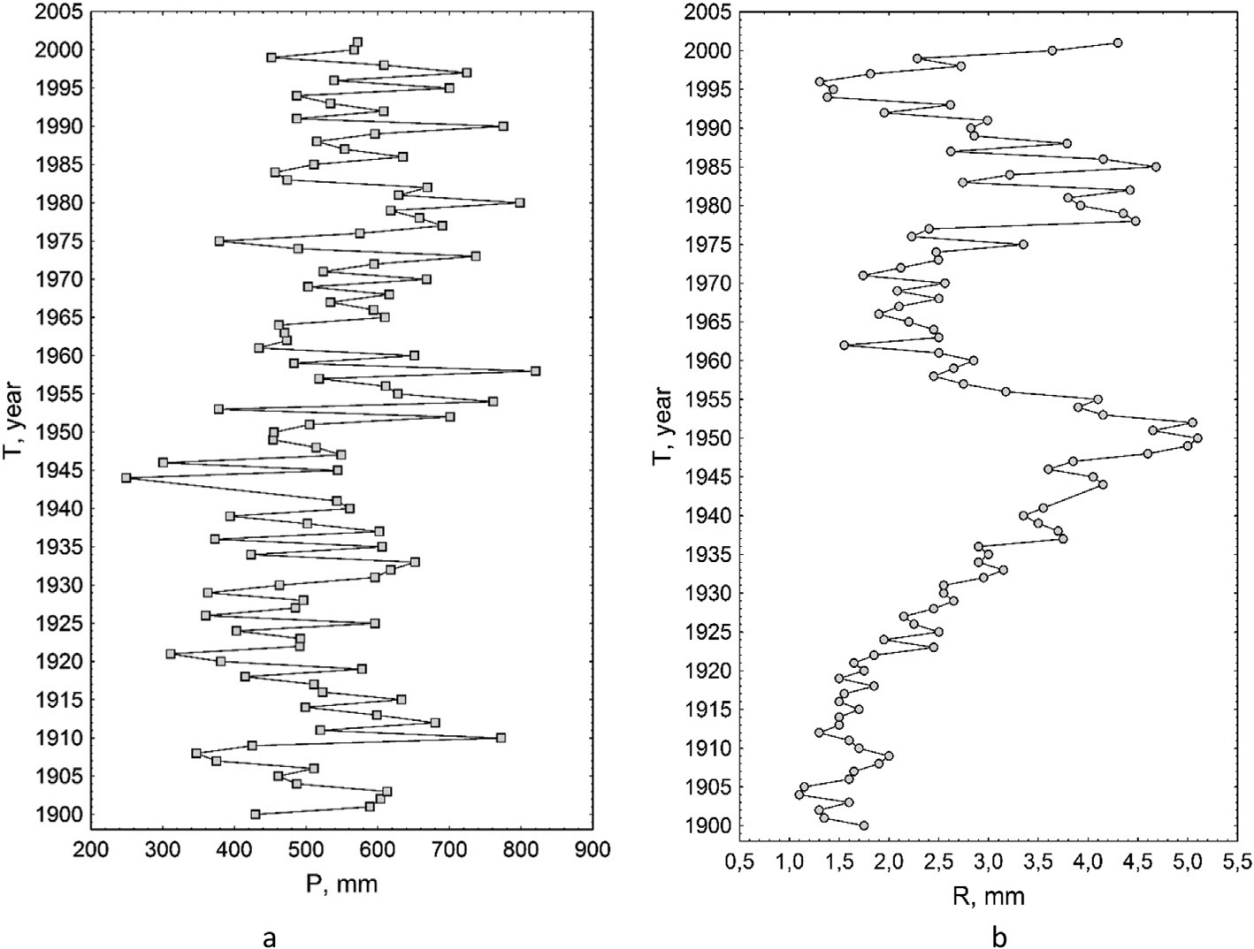
The initial time series of changes in precipitation (meteorological station ‘Bogoroditskoye-Fenino’) (a) and tree-ring growth of the oak (b).

Table 1 shows the ratio of content of chemical elements in particles <1 mm and soil sample. Table 2 contains concentration of the macroelements and trace elements in a transect-catena and in the pedosediments. Table 3 contains values of geochemical relationships and coefficients with coefficient of variation (V) values of less than 10%. Fig. 1 shows the vertical profile of pedosediments and their lower border. Fig. 2, Fig. 3 shows profile distribution of the main physico-chemical and geochemical parameters. Fig. 4 shows the results of a cluster analysis based on the concentration of chemical elements and oxides in the layers of pedosediments (2019). Fig. 5 shows the results of a cluster analysis by the values of geochemical coefficients in the layers of pedosediments (2019). Fig. 6 shows the initial time series of changes in precipitation and growth of tree rings that were used for palaeogeographic reconstructions. Source data is available in the repository [8].

**Table 1:** The coefficient of selectivity (Кsi) as ratio of content of chemical elements in particles <1 mm and soil sample.

No	Catena on the slope of the rampart									Bottom of the ditch			Pedosediments (2013 year)							
	1–1	1–2	1–3	2–1	2–2	2–3	3–1	3–2	3–3	4–1	4–2	4–3	5–1	5–2	5–3	5–4	5–5	5–6	5–7	5–8
Layer, cm	0–14	14–20	20–30	0–10	10–20	20–30	0–10	10–20	20–30	0–10	10–20	20–30	0–10	10–20	20–30	30–50	50–70	70–90	90–95	95–100
SiO^2^	1.08	0.95	1.01	0.94	1.04	0.99	0.97	0.89	0.95	1.02	0.96	1.00	0.98	0.92	1.04	1.04	0.96	0.94	0.96	1.02
Al_2_O_3_	1.00	0.96	0.99	0.97	0.98	1.01	0.97	0.90	0.92	0.95	0.93	0.96	0.97	0.90	1.02	0.99	0.92	0.90	0.98	1.03
CaO	1.01	1.03	0.97	0.97	1.08	0.99	1.03	1.09	0.95	1.02	0.94	0.98	0.98	1.03	0.98	1.01	1.02	1.05	1.03	0.98
Fe	1.07	0.94	1.01	0.97	1.00	1.03	0.95	0.97	0.99	1.00	1.00	0.96	1.03	0.98	0.97	1.00	1.00	1.00	1.02	0.97
TiO2	1.03	1.02	1.06	0.94	0.99	1.05	0.94	0.92	0.96	0.99	1.04	0.96	0.98	0.94	1.01	1.02	0.96	0.96	0.98	0.90
K_2_O	1.04	0.96	1.01	0.94	0.97	0.95	0.93	0.91	0.88	0.99	0.93	1.00	0.95	0.97	0.97	1.03	0.97	0.95	0.96	0.97
MgO	1.11	1.05	0.98	0.91	1.00	0.98	1.01	0.88	0.90	0.90	0.95	0.94	1.04	0.92	1.06	0.98	0.88	0.98	1.00	1.08
Na_2_O	1.21	1.38	0.62	1.11	1.16	0.86	1.07	0.72	1.34	0.95	0.50	1.00	0.98	0.82	0.76	1.09	1.33	0.98	0.99	1.42
P_2_O_5_	1.05	1.03	1.00	0.99	1.08	1.02	1.00	0.99	0.96	1.02	0.98	0.99	0.96	1.04	1.07	1.11	1.08	1.00	1.08	0.98
MnO	1.05	1.06	1.15	0.93	0.94	1.05	1.02	0.96	0.95	0.99	1.01	0.96	0.99	0.96	1.02	0.99	0.93	0.92	0.97	0.98
As	0.89	1.17	0.91	1.13	1.08	1.02	1.03	0.87	0.98	0.87	0.78	0.81	1.08	0.91	0.96	1.00	1.02	1.04	0.90	1.07
Ba	1.08	1.00	1.03	0.96	1.04	1.05	0.96	0.98	1.00	1.00	0.96	0.95	1.05	1.02	0.98	0.97	1.02	0.98	1.04	0.98
Co	0.51	1.11	1.26	0.86	0.73	1.05	0.92	0.85	0.74	0.92	1.23	1.04	0.70	0.84	0.88	0.91	0.95	0.97	0.71	0.96
Cu	1.10	0.93	1.06	0.97	1.09	1.02	0.99	1.07	0.98	0.96	1.12	1.02	0.99	1.09	0.99	0.95	0.85	0.98	1.03	0.99
Cr	1.09	0.97	0.97	0.97	0.98	1.14	0.93	0.97	1.05	1.06	1.20	1.04	1.07	1.08	1.01	1.08	1.18	0.95	1.04	0.81
Ni	1.07	0.96	0.99	1.03	1.00	1.01	0.99	0.99	0.99	0.97	0.99	0.96	1.03	1.00	1.01	0.96	1.01	0.96	0.98	0.95
V	1.19	0.87	1.02	0.93	1.12	1.13	0.89	1.04	1.15	1.09	1.06	1.01	1.14	1.10	0.87	0.95	1.11	0.97	1.13	0.93
Pb	1.05	0.62	1.10	0.76	0.90	1.09	1.01	1.45	1.09	1.21	0.86	0.97	1.04	0.92	0.90	0.67	0.75	0.76	1.08	0.79
Rb	1.05	0.93	1.00	0.99	0.99	1.06	0.98	0.96	0.96	0.97	0.98	0.95	1.04	1.01	0.97	0.98	1.03	0.99	1.00	0.97
Sr	1.36	1.02	1.05	0.93	1.12	1.08	0.96	1.13	1.21	1.23	1.01	0.91	1.27	1.00	0.91	0.91	1.29	1.19	1.32	0.97
Zn	0.91	1.03	0.96	1.03	0.89	0.91	1.05	0.96	0.97	0.96	0.88	0.85	0.92	0.92	1.05	0.93	1.01	0.96	0.83	1.00

**Table 2:** Soil colour, content of the macroelements and trace elements in a transect-catena in the bottom of the ditch and in the pedosediments.

Point-layer no.			1	3	4	5–1	5–2	5–3	5–4	5–5	5–6	5–7	5–8	5–9	5–10
Colour: moist (10YR)			2/1	3/1.5	3/1	3/2	3/1.5	3/1	3/2	3/1	3/1	3/1.5	3/3	4/3	5/6
Colour: dry (10YR)			3/1	3/2	3/1.5	3/2	3/1.5	3/2	3/2.5	3/2	3/2	3/3	5/3	5/3	5/4
Macro-elements (%)		SiO_2_	58.0	57.0	61.1	55.9	53.6	57.8	53.1	52.0	49.1	56.3	53.7	56.4	52.5
		Al_2_O_3_	9.8	10.0	9.0	9.7	9.2	9.5	9.3	9.1	9.3	8.7	10.0	10.2	10.4
		CaO	1.6	1.4	1.3	1.5	1.4	1.6	1.5	1.5	1.3	1.4	1.5	4.0	2.4
		Fe	2.9	3.0	2.6	2.7	3.0	2.9	3.0	3.0	3.0	2.8	3.1	2.8	3.1
		TiO_2_	0.8	0.8	0.7	0.7	0.8	0.7	0.8	0.7	0.7	0.7	0.7	0.7	0.8
		K_2_O	2.0	2.1	2.1	1.9	1.9	2.0	1.9	1.9	1.8	1.9	1.8	1.8	1.6
		MgO	0.7	0.8	0.7	0.7	0.8	0.8	0.7	0.7	0.7	0.5	0.8	1.0	0.8
		Na_2_O	0.6	0.8	0.7	0.7	0.6	0.6	0.9	0.7	0.6	0.7	0.6	0.7	0.9
		P_2_O_5_	0.1	0.1	0.1	0.1	0.1	0.1	0.1	0.1	0.1	0.1	0.1	0.1	0.1
Trace elements (ppm)		MnO	0.1	0.1	0.1	0.1	0.1	0.1	0.1	0.1	0.1	0.1	0.1	0.1	0.0
		Zr	393.2	424.2	393.2	415.5	445.6	419.7	436.8	438.0	416.1	435.7	428.2	375.7	434.1
		Ba	466.2	489.1	464.5	434.2	479.3	461.4	479.6	492.8	473.4	479.5	458.6	421.4	420.5
		Sr	102.7	99.6	92.7	78.1	110.2	96.9	111.6	106.8	90.9	119.9	95.0	91.7	89.5
		V	107.6	104.4	96.6	95.5	104.4	107.8	109.6	107.1	107.9	98.5	114.7	103.4	110.4
		Cr	90.5	101.6	91.3	86.4	90.4	93.7	92.7	97.9	97.4	92.8	102.6	87.7	93.7
		Rb	85.4	83.8	81.5	82.6	86.8	85.2	87.9	87.9	78.2	84.6	83.8	73.5	81.1
		Zn	58.7	60.2	59.9	56.6	56.3	54.6	54.9	54.9	69.8	53.5	53.9	54.0	53.5
		Cu	43.8	44.9	38.1	45.1	44.1	41.6	46.7	47.3	53.2	41.0	51.0	41.8	46.5
		Ni	41.5	42.2	34.1	38.8	41.8	40.1	42.8	43.7	45.3	37.7	44.8	39.7	43.8
		Pb	21.7	18.0	22.5	19.4	16.2	17.8	20.9	17.4	20.8	19.1	18.7	17.2	18.9
		Co	12.6	11.7	12.0	11.8	15.7	15.2	11.2	12.8	10.2	15.3	13.1	11.1	14.2
		As	7.2	6.5	7.2	7.1	7.1	7.2	6.1	6.6	6.2	6.8	7.0	6.7	5.9

Point-layer no.: 1 – top of the rampart; 3 – middle of the ditch; 4 – mouth of the ditch; 5 – vertical profile of the pedosediments.

**Table 3:** Values of geochemical relationships and coefficients with coefficient of variation (V) values of less than 10%.

Geochemical relationships and coefficients	Numbers of layers in pedosediments of the ditch (according to Fig. 1)								V, %
	5–1	5–2	5–3	5–4	5–5	5–6	5–7	5–8	
SiO_2_/(Al_2_O_3_+Fe + MnO)	4.68	4.51	4.50	4.47	4.43	4.26	4.34	4.36	3
SiO_2_/(CaO + MgO + K_2_O + Na)	13.38	13.57	11.99	13.31	14.33	14.29	13.99	14.10	6
(Na + K_2_O + MgO + Zn)/SiO_2_	1.25	1.22	1.21	1.18	1.08	1.20	1.15	1.14	5
(SiO_2_+Al_2_O_3_)/Fe	26.14	25.27	23.45	27.76	27.08	25.63	24.56	24.47	6
SiO_2_/Al_2_O_3_	5.96	5.74	5.84	5.55	5.52	5.34	5.52	5.54	4
(Fe + MnO)/Al_2_O_3_	0.27	0.27	0.30	0.24	0.25	0.25	0.27	0.27	7
TiO_2_/Al_2_O_3_	0.07	0.07	0.08	0.06	0.07	0.07	0.07	0.07	6
Ti/(Al_2_O_3_+CaO + Na + K_2_O)	0.05	0.05	0.05	0.05	0.05	0.05	0.05	0.05	4
Zr/TiO_2_	496.97	533.33	506.17	525.28	483.12	484.53	524.22	508.61	4
Zr/(Al_2_O_3_+CaO + Na + K_2_O)	25.77	28.30	27.08	24.54	24.24	25.03	27.56	27.24	6
MnO/Al_2_O_3_	0.01	0.01	0.01	0.01	0.01	0.01	0.01	0.01	8
MnO/Fe	0.02	0.02	0.02	0.02	0.02	0.02	0.02	0.02	6
Al_2_O_3_/(CaO + Na + K_2_O + MgO)	2.25	2.36	2.05	2.40	2.60	2.67	2.54	2.54	8
(K_2_O + Na_2_O)/Al_2_O_3_	0.23	0.22	0.24	0.20	0.19	0.19	0.21	0.20	9
(CaO + MgO + K_2_O)/Al_2_O_3_	0.41	0.38	0.44	0.38	0.36	0.35	0.35	0.37	8
Ke’ = Ti/(Ca + K + Mg + Na)	13.20	13.37	11.83	13.13	14.14	14.08	13.79	13.91	6
Ke” = Al/(Ca + K + Mn + Mg + Na)	2.21	2.33	2.03	2.37	2.56	2.64	2.50	2.51	8
Σ HM = (Ni + Cu + Pb + Cr + As + V)	270.59	271.12	278.07	273.33	270.49	283.93	283.08	303.93	4
Σ BIO = (CaO + Al_2_O_3_+MnO + Fe + SiO_2_+K_2_O + MgO + Ni + Cu + Zn)	237.84	232.65	223.45	241.54	237.54	241.58	235.92	235.99	2
Σ BIO/Fe	87.40	83.04	79.16	88.40	84.49	84.34	80.48	79.25	4
Σ BIO/Al_2_O_3_	23.27	22.14	23.10	20.85	20.34	20.88	21.36	21.20	5

## 2 Experimental design, materials, and methods

### 2.1 Physico-chemical properties of soils and pedosediments

The granulometric analysis was conducted using laser analyser of the size of particles Analysette 22 MicroTec. Sample preparation of dry soil was carried out by sieving through sieves with a mesh size of 0.25 mm. The preparation of soil samples for chemical analyses was fulfilled in two variants: reduction of the entire weighted sample of the soil to a powdered state and applying of this procedure only to structural units sized <1 mm. Through the ratio of the content of each chemical element (1, 2, …i) in particles measuring <1 mm and over the entire soil sample, an indicator was calculated which was called the coefficient of selectivity (Кsi) (Table 1). Chemical analyses of soils (Table 2) included the following standard procedures: assessment of the CO_2_ in carbonates by acidometer; the pH values (H_2_O) were determined by a potentiometric method. Colours (dry/moist) were described using the Munsell-System [9]. Distribution by profile of pedosediments of the main physico-chemical parameters, which were determined in 2013 are shown in Fig. 2.

### 2.2 Geochemical data on soils and pedosediments

Wavelength-dispersion X-ray fluorescence spectrometer was used to determine the contents of chemical elements. Concentrations of macroelements and trace elements in soils (25 metals and oxides) were determined by the technique of measuring metal mass fraction and oxides in powdered samples. Quantitative calibration was performed using a set of State standard samples of soil composition. The results that were obtained for the first and second working samples were averaged, and the acceptability of the results was checked by comparing their discrepancies with the standard (the permissible discrepancy between the results under repeatability conditions for probability P = 0.95).

### 2.3 Geochemical relationships and coefficients

Using the determinations of the bulk composition of soils and sediments, the most informative geochemical indicators were calculated: CaO/TiO_2_, CaO/ZrO_2_, Si/(Al + Fe + Mn), (CaO + MgO)/Al_2_O_3_, SiO_2_/Al_2_O_3_, the sum of the elements accumulated in the soil (P, Ca, K, Mg, Mn, Cu) [10] – S_A_. Accepting Liu's proposition [11] concerning the eluviation coefficient (Ke), the modified variant of the formula included the basic oxides: Ке = (SiO_2_/(MnO + CaO + K_2_O + MgO + Na_2_O)). A geochemical classification of the elements in terms of the peculiarity of their migration was employed by А.I. Perel'man [10] enabling to evaluate the landscape characteristics of the association of mobile (Ca, Na, Mg, Sr) and weakly mobile (K, Ba, Rb) elements – Σmob. All these informative indicators had a coefficient of variation (V) of more than 18%, in contrast to other tested geochemical relationships and coefficients (Table 3). Two curves of the profile distribution of geochemical relationships and coefficients according to the results of testing by layers of 2013 (cm) and 2019 (cm) are presented in Fig. 3.

### 2.4 Hierarchical classification of layers of pedosediments

Grouping of the strata of pedosediments was conducted by the method of the hierarchic classification (unification by Ward's method) of cluster analysis according to the most informative indices normalized through the mean-square deviation. The concentration of those of chemical elements and oxides that were used to calculate geochemical coefficients are presented in Fig. 4.

2019 data on ten geochemical coefficients for which the coefficient of variation is V> 18% (content of particles (%) with the size >0.01 and < 0.005 mm, pH_H2O_, CO_2,_ (CaО+MgO):Al_2_O_3,_ Si:Al, CaO:TiO_2,_ S_A,_ Кe, Σmob), were used for the hierarchical classification of the pedosediment layers (Fig. 5).

### 2.5 Definition of the rhythms of precipitation and tree-ring growth of the oak-tree

The initial data of precipitation from the meteorological station ‘Bogoroditskoye-Fenino’ (Fig. 6, a) and tree-ring growth of the oak (Fig. 6, b) were used for palaeogeographic reconstruction (going beyond the instrumental period). The synchronism of the rhythms of precipitation and tree-ring growth was investigated by means of the method of difference integral curves of modular coefficients. The cyclic constituents of the dendro-chronoseries and precipitations were established by means of Fourier analysis. Filtration of the noises in the rhythms of many years was conducted using 4253H filter, which allowed us to preserve the main rhythmic characteristics of the original one. Time series processing was performed using the licensed software STATISTICA Advanced + QC for Windows v.10 Ru and STATISTICA Automated Neural Networks for Windows v.10 Ru were employed.

### 2.6 Data analysis

The data were represented a system of most information-rich pedogenetic indicators for reconstruction of the dynamics of erosion-accumulative processes on the basis of dated earthen defensive constructions of the historical period (last 367 years). The prospect of using the presented data is to supplement the created databases of objects of historical and cultural heritage [12,13] with the results of soil and geochemical studies of the catenas and pedosediments.
